# Supplementary material for: Native-like SARS-CoV-2 spike glycoprotein expressed by ChAdOx1 nCoV-19/AZD1222 vaccine
Source: bioRxiv. 2021 Jan 19:2021.01.15.426463. Preprint. [Version 1] doi: 10.1101/2021.01.15.426463 (PMC7836103; doi:10.1101/2021.01.15.426463)
Supplement: Supplement 1 [file media-1.pdf]

Supplementary Information for:

Native-like SARS-CoV-2 spike glycoprotein expressed by ChAdOx1 nCoV-19/AZD1222 vaccine

Yasunori Watanabe[1,2*], Luiza Mendonça[3*], Elizabeth R. Allen[4*], Andrew Howe[5], Mercede Lee[4], Joel D. Allen[1], Himanshi Chawla[1], David Pulido[4], Francesca Donnellan[4], Hannah Davies[4], Marta Ulaszewska[4], Sandra Belij-Rammerstorfer[4,6], Susan Morris[4], Anna-Sophia Krebs[3], Wanwisa Dejnirattisai[7], Juthathip Mongkolsapaya[7,9,10], Piyada Supasa[7], Gavin R. Screaton[7,8], Catherine M. Green[7], Teresa Lambe[4,6,#], Peijun Zhang[3,5,#], Sarah C. Gilbert[4,6,#], Max Crispin[1,#]

[1]School of Biological Sciences, University of Southampton, Southampton, SO17 1BJ, UK

[2]Oxford Glycobiology Institute, Department of Biochemistry, University of Oxford, South Parks Road, Oxford, OX1 3QU, UK

[3]Division of Structural Biology, University of Oxford, Wellcome Centre for Human Genetics, Oxford, OX3 7BN, UK

[4]The Jenner Institute, Nuffield Department of Medicine, University of Oxford, Oxford, UK

[5]Electron Bio-imaging Centre, Diamond Light Source, Harwell Science and Innovation Campus, Didcot, OX11 0DE, UK

[6]NIHR Oxford Biomedical Research Centre, Oxford, UK

[7]The Wellcome Centre for Human Genetics, University of Oxford, Roosevelt Drive, Oxford OX3 7BN, UK

[8]Division of Medical Sciences, John Radcliffe Hospital, University of Oxford, Oxford, UK

[9]Dengue Hemorrhagic Fever Research Unit, Office for Research and Development, Faculty of Medicine, Siriraj Hospital, Mahidol University, Bangkok, Thailand

[10]Chinese Academy of Medical Science(CAMS) Oxford Institute (COI), University of Oxford, Oxford, U.K.

[#]Corresponding authors. Email: max.crispin@soton.ac.uk (M.C.), peijun@strubi.ox.ac.uk (P.Z), sarah.gilbert@ndm.ox.ac.uk (S.C.G.), teresa.lambe@ndm.ox.ac.uk (T.L.)

*These authors contributed equally to this work

This document includes:

- Supplementary Figures 1-4
- Supplementary Table 1

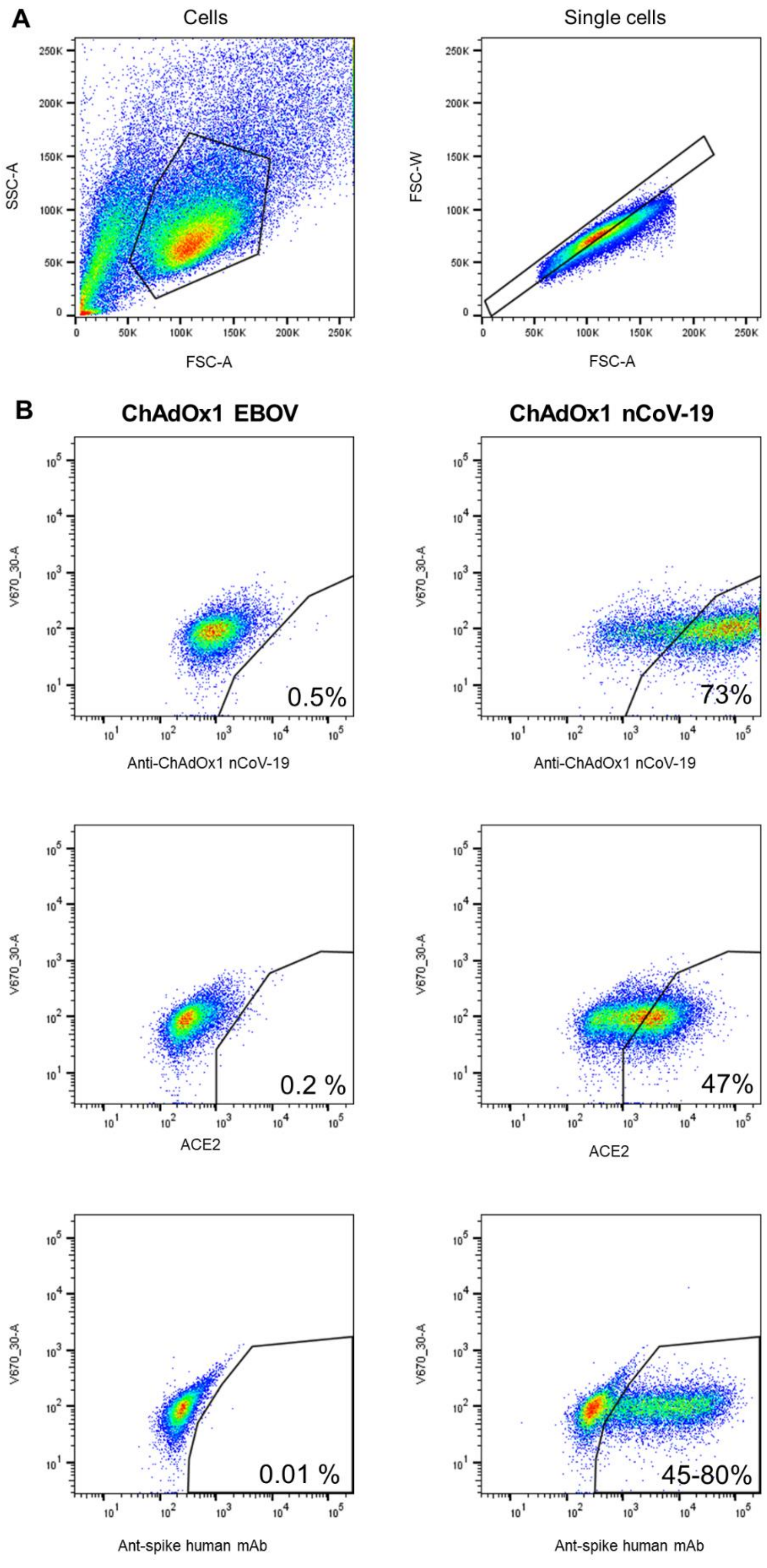

Supplementary Figure 1. Gating strategy for vaccine expression FACS

(A) Single cells were isolated for further analysis. **(B)** Percentages of positive cells were determined using the gating strategy determined in uninfected cells and displayed for ChAdOx1- EBOV (left panels) and ChAdOx1-nCoV19 (right panels) infected cells, with percentage of positive cells indicated in gate. Representative data is shown, for human mAbs data for mAb 71 is shown.

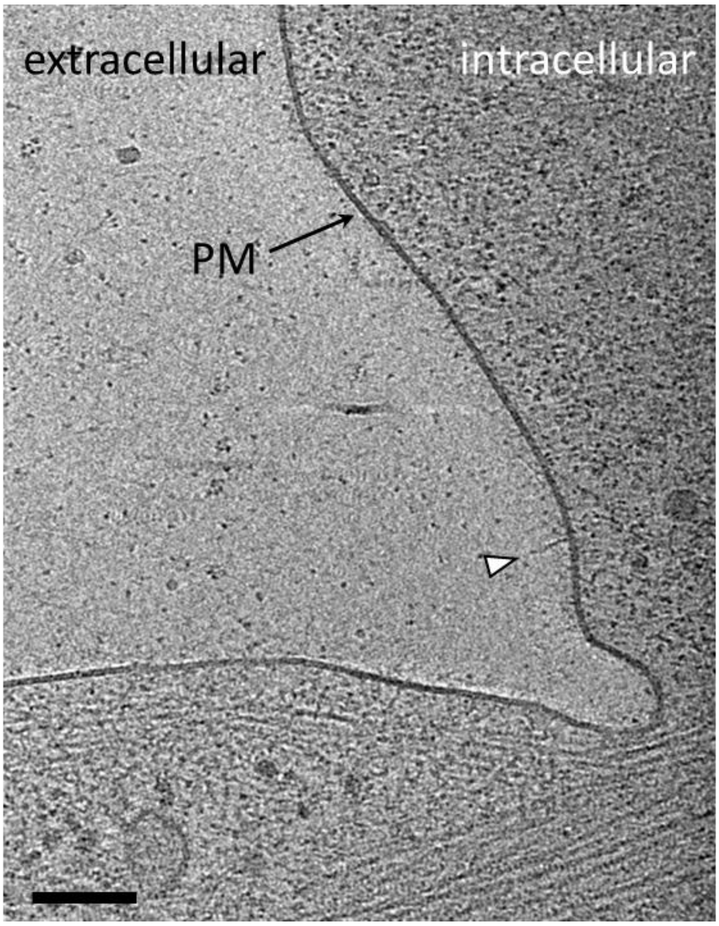

Supplementary Figure 2. Non-transduced cells lack prefusion-like surface receptors. Tomographic slice of U2OS control cell surface. PM − Plasma membrane. Slice is 2.13 Å thick. White arrowhead point to a thin and elongated surface receptor. Scale bar is 100 nm.

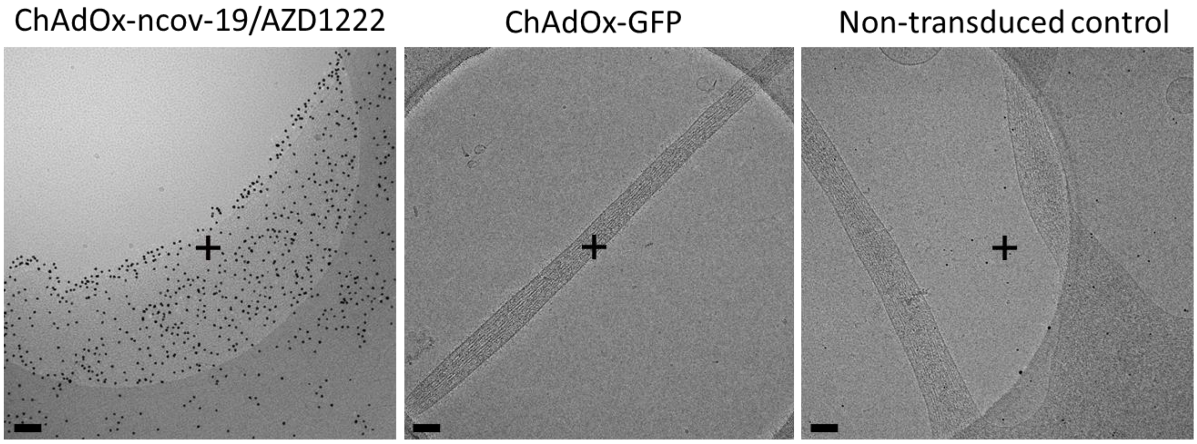

Supplementary Figure 3. Cryoimmunolabelling of ChAdOx-nCoV-19/AZD1222 derived spike. Cryo-EM image of U2OS cells transduced with ChAdOx-nCoV-19/AZD1222, ChAdOx-GFP and non-transduced controls. Cells were incubated with ChAdOx-nCoV-19/AZD1222 vaccinated mice sera and labelled with anti-mouse Fab conjugated with 10nm Au beads prior to plunge freezing. Scale bar is 100 nm.

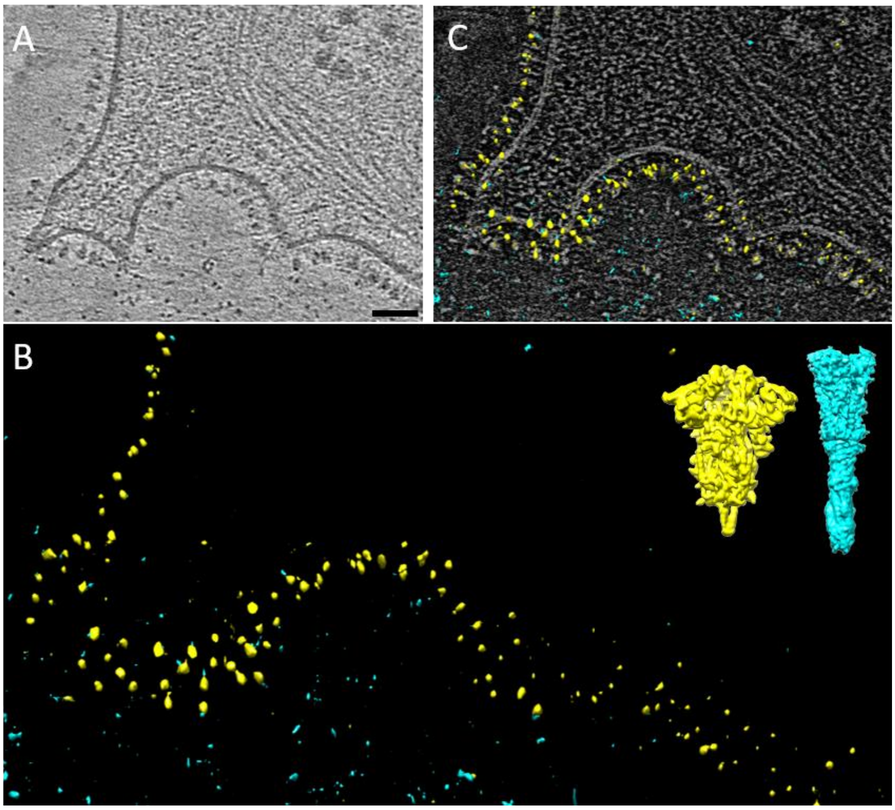

Supplementary Figure 4. Template searching for pre- and post-fusion spike proteins. (A) Tomographic slice of a ChAdOx nCoV-19 infected U2OS cell. (B) Results of template matching by cross-correlation of tomographic volume with either a pre-fusion spike reference (EMD-21452, yellow) or a post-fusion spike reference (EMD-7040, cyan). The resulting cross-correlation maps are projected through 18.5 nm thick volume. Inset, the pre- (yellow) and post-fusion (cyan) spike references. (C) The template matching results shown in (B) overlaid with a 1.6 nm thick tomographic slice in (A). Scale bar is 50 nm.

Supplementary Table 1. Glycoform abundances observed across S0 and S1/S2 SARS-CoV-2 spike protein derived from ChAdOx1 nCoV-19.

Cleaved

	N17	N61	N74	N122	N149	N165	N234	N282	N331	N343	N603	N616	N657	N709	N717	N801	N1074	N1098	N1134	N1158	N1173	N1194	Total
Mannose/Hybrid	0%	81%	0%	60%	n/a	0%	94%	18%	11%	74%	0%	100%	100%	100%	100%	82%	100%	25%	n/a	n/a	n/a	n/a	56%
Complex	0%	19%	100%	40%	n/a	100%	6%	82%	89%	26%	100%	0%	0%	0%	0%	18%	0%	75%	n/a	n/a	n/a	n/a	38%
Unoccupied	100%	0%	0%	0%	n/a	0%	0%	0%	0%	0%	0%	0%	0%	0%	0%	0%	0%	0%	n/a	n/a	n/a	n/a	6%

Uncleaved

	N17	N61	N74	N122	N149	N165	N234	N282	N331	N343	N603	N616	N657	N709	N717	N801	N1074	N1098	N1134	N1158	N1173	N1194	Total
Mannose/Hybrid	n/a	100%	100%	81%	n/a	72%	98%	74%	78%	69%	69%	100%	100%	100%	100%	66%	86%	69%	100%	n/a	n/a	73%	85%
Complex	n/a	0%	0%	19%	n/a	28%	2%	26%	22%	31%	31%	0%	0%	0%	0%	34%	14%	31%	0%	n/a	n/a	27%	15%
Unoccupied	n/a	0%	0%	0%	n/a	0%	0%	0%	0%	0%	0%	0%	0%	0%	0%	0%	0%	0%	0%	n/a	n/a	0%	0%
